# Protons Sensitize Epithelial Cells to Mesenchymal Transition

**DOI:** 10.1371/journal.pone.0041249

**Published:** 2012-07-23

**Authors:** Minli Wang, Megumi Hada, Janapriya Saha, Deepa M. Sridharan, Janice M. Pluth, Francis A. Cucinotta

**Affiliations:** 1 Division of Space Life Sciences, Universities Space Research Association, Houston, Texas, United States of America; 2 Department of Cancer and DNA Damage Responses, Lawrence Berkeley National Laboratory, Berkeley, California, United States of America; 3 Space Radiation Program, Lyndon B. Johnson Space Center, National Aeronautics and Space Administration (NASA), Houston, Texas, United States of America; National Taiwan University, Taiwan

## Abstract

Proton radiotherapy has gained more favor among oncologists as a treatment option for localized and deep-seated tumors. In addition, protons are a major constituent of the space radiation astronauts receive during space flights. The potential for these exposures to lead to, or enhance cancer risk has not been well studied. Our objective is to study the biological effects of low energy protons on epithelial cells and its propensity to enhance transforming growth factor beta 1 (TGFβ1)-mediated epithelial-mesenchymal transition (EMT), a process occurring during tumor progression and critical for invasion and metastasis. Non-transformed mink lung epithelial cells (Mv1Lu) and hTERT- immortalized human esophageal epithelial cells (EPC) were used in this study. EMT was identified by alterations in cell morphology, EMT-related gene expression changes determined using real-time PCR, and EMT changes in specific cellular markers detected by immunostaining and western blotting. Although TGFβ1 treatment alone is able to induce EMT in both Mv1Lu and EPC cells, low energy protons (5 MeV) at doses as low as 0.1 Gy can enhance TGFβ1 induced EMT. Protons alone can also induce a mild induction of EMT. SD208, a potent TGFβ Receptor 1 (TGFβR1) kinase inhibitor, can efficiently block TGFβ1/Smad signaling and attenuate EMT induction. We suggest a model for EMT after proton irradiation in normal and cancerous tissue based on our results that showed that low and high doses of protons can sensitize normal human epithelial cells to mesenchymal transition, more prominently in the presence of TGFβ1, but also in the absence of TGFβ1.

## Introduction

Proton irradiation has grown in importance as a modality for cancer treatment in part because of its ability to spare normal tissues as compared to conventional radiation [1], [2]. Nearly 100,000 patients have been treated with protons for prostate, esophageal, lung, head and neck, and other cancers, and there are a growing number of proton treatment centers worldwide that will operate in the near future. Enhanced sparring of tissue is possible by careful placement within the tumor volume of the proton peak energy deposition, denoted as the Bragg peak, which occurs as a particle slows down before it comes to rest. The close proximity of the esophagus to other tissues such as the heart, lung and spinal cord, makes the treatment of esophageal cancer difficult, thus proton therapy can be an excellent choice for treating patients with esophageal cancer [3]. In addition, protons are a main constituent of the space radiation astronauts receive during space flights [4]. Given that some proton exposure will still occur in tissue near the edges of the tumor, and the possibility that high linear energy transfer (LET) protons (LET>10 keV/µm) may produce qualitatively different biological effects compared to low LET radiation such as x-rays [4], it is important to study how protons may specifically affect the cells and tissues.

Epithelial-mesenchymal transition (EMT), long recognized as a cellular mechanism in embryonic development, has been associated with wound healing, fibrosis of heart, lung, liver and kidney [5], and more recently studies have linked this process to cancer progression and the generation of cancer stem cells [6]–[8]. EMT involves cell morphology alterations, cytoskeletal rearrangements, reduced cell adhesion and increased motility, and resistance to apoptosis. During EMT, epithelial characteristics are lost with nuclear translocation of β-catenin and loss of E-cadherin and there is a gain of expression of mesenchymal markers, such as fibronectin and vimentin [9].

EMT can be initiated by a variety of cytokines and stimuli, including exposure to transforming growth factor beta1 (TGFβ1), which is a main regulator of EMT. TGFβ1 is a multifunctional cytokine that modulates cell proliferation, differentiation, apoptosis, and extracellular matrix production [10], [11]. TGFβ1 exerts its effects through Smad-dependent and Smad-independent pathways [10], [11]. Elevated expression of TGFβ1 has been observed in patients with breast, lung, ovarian, cervical and esophageal cancers [12], [13]. Therefore increased expression of TGFβ1 in cancerous tissues may trigger the process of EMT promoting invasion and tumor progression. Radiation can cause several alterations, including DNA damage, oxidative stress, apoptosis, senescence, and genomic instability, which may lead to carcinoma or cell death. Radiation has been shown to lead to the activation of the inactive form of TGFβ1, LAP-TGFβ1 (LAP, Latency-associated Peptide). A rapid activation of latent TGFβ1 by radiation-induced reactive oxygen species has been found in mouse mammary glands [14], and detectable levels of TGFβ1 have been shown to persist up to 2 weeks following ion exposure [15], suggesting TGFβ1 activation is one of the most responsive indices of tissue exposure to ionizing radiation (IR). Although the effects of TGFβ1 on EMT in cancerous tissues has been studied to some extent; much less is known about its effects in normal tissue, and the combined effects of radiation and TGFβ1 on normal or cancerous tissues.

**Figure 1:**
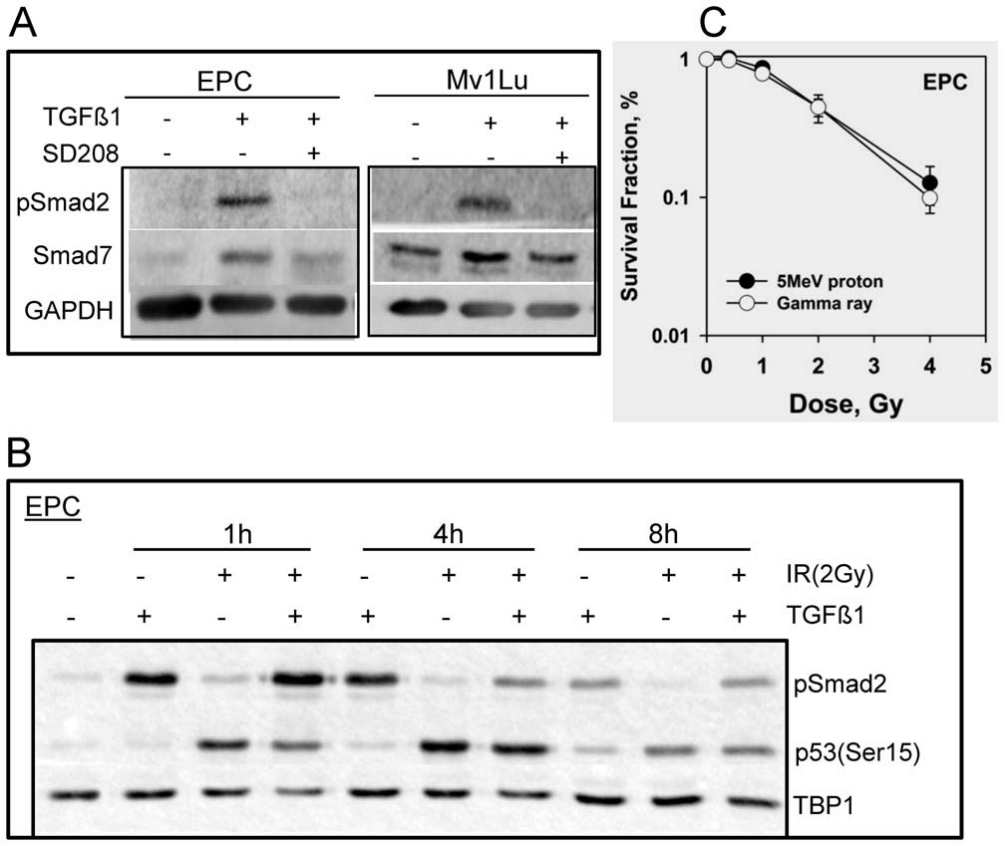
Cellular response to TGFβ1 and IR exposure in EPC and Mv1Lu cells. A. Whole cell extracts were prepared 4 h after 2 ng/ml TGFβ1 treatment using M-PER kit. Phosphorylation of Smad2 and expression of Smad7 were detected using western blotting. TGFβR1 inhibitor- SD208 (or DMSO as control) was added one hour prior to the addition of TGFβ1. GAPDH was used as loading control. B. Nuclear extracts were isolated from EPC cells using NE-PER kit after 2 Gy of proton radiation at indicated times, in the presence or absence of TGFβ1. Shown are the western blots with phosphorylation of Smad2 and p53, and TBP1 as internal loading control. C. Clonogenic survival assay of EPC cells with different doses of protons and gamma rays as described in Materials and Methods.

To elucidate the advantages of proton radiotherapy in the treatment of cancer, there is a critical need to study the possible risks of this exposure to normal tissue. In this study, we investigated the potential of protons exposure, both in the presence of elevated TGFβ1 and in its absence to elicit EMT in exposed cells. Two cell lines: mink lung epithelial (Mv1Lu) cells-a known TGFβ1-sensitive non-transformed cell line [16], and human hTERT immortalized esophageal epithelial (EPC) cells were used in this study. We first demonstrated the effect of TGFβ1 on Smad signaling pathways, including the phosphorylation of Smad2, and up regulation of inhibitory Smad7. Conventional immunostaining or immunoblotting of proteins known to be altered in EMT were used to detect changes upon TGFβ1 stimulation post protons exposure at high and low doses. Relative changes in the expression of EMT related genes were analyzed using real-time PCR.

**Figure 2:**
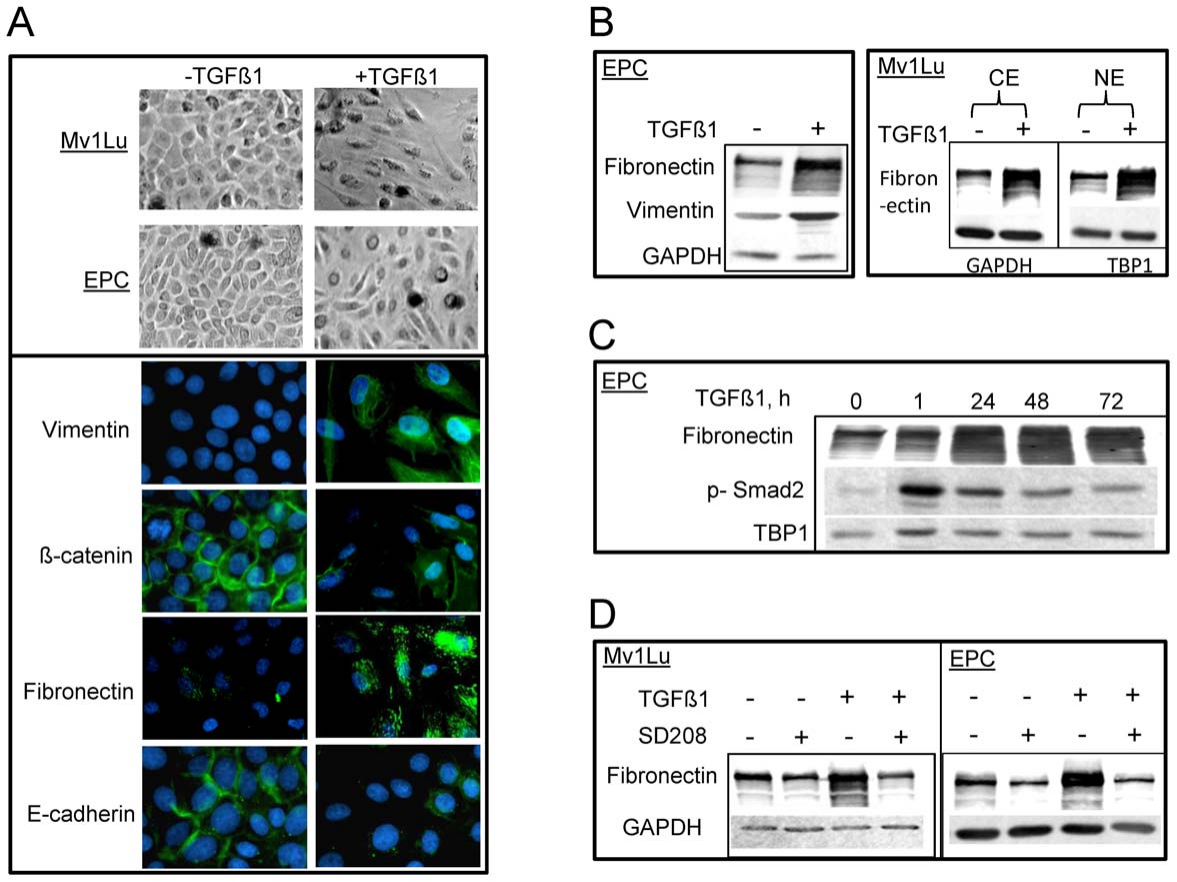
TGFβ1 induces EMT in both EPC and Mv1Lu cells. A. Phase contrast images of morphology alterations after TGFβ1 stimulation in EPC and Mv1Lu cells. Hallmarks for mesenchymal phenotype including vimentin, β-catenin, E-cadherin and fibronectin (green immunofluorescence) are shown in EPC cells 3 days after TGFβ1 treatment (right, lower panel). DAPI staining (blue) represents cell nuclei. B. The expression of EMT markers using western blotting from whole cells extracts in EPC cells. Right upper panel shows expression of fibronectin in both cytoplasmic extract and nuclear extract in the presence or absence of TGFβ1treatment (2 days) in Mv1Lu cells. GAPDH and TBP1 were used as loading control of cytoplasmic extract and nuclear extract, respectively. C. Expression of fibronectin and p-Smad2 in nuclear extract of EPC cells after different incubation time with TGFβ1. D. Effect of inhibition of fibronectin expression upon TGFβ R1 inhibitor -SD208 treatment in both EPC and Mv1Lu cells. Whole cell extracts were used for western blotting. Other details were shown in Figure 1.

## Results

### Prompt cellular response to TGFβ1 and proton radiation was observed in epithelial cells

We first analyzed the TGFβ1/Smad signaling in response to TGFβ1 as well as its inhibition in the presence of the novel TGFβR1 inhibitor-SD208. Phospho-Smad2 (pSmad2) was induced promptly after TGFβ1 addition, both in Mv1Lu and EPC cells (Fig. 1A). The expression of Smad7 was increased in concert with Smad2 phosphorylation. As expected, pretreatment with the TGFβR1 inhibitor, altered pSmad2 expression and attenuated Smad7 expression. Smad2 phosphorylation was highest in EPC cells at 1 hour post the treatment (Fig. 1B) and decrease dramatically after 8 hours.

**Figure 3:**
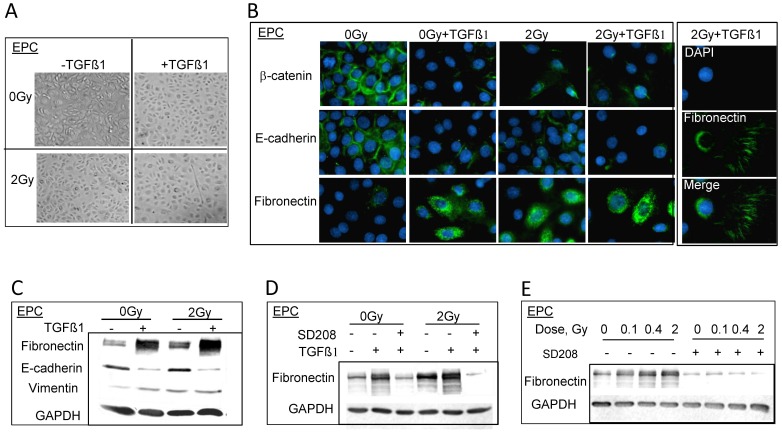
Protons enhance EMT induced by TGFβ1 in EPC cells. A. Phase contrast images of morphology alterations after double treatment of TGFβ1 and 2 Gy of protons. B. Immunostaining of EMT markers, 3 days post 2 Gy of protons in the presence or absence of TGFβ1. Right panel shows the tracks of fibronectin staining in EPC cells after double treatment. C. Western blots show the change of EMT markers expression upon TGFβ1 and/or protons treatment. Whole cell extracts were prepared using RIPA buffer. D. Western blots show the inhibition of fibronectin expression when EPC cells were pretreated with TGFβR1 inhibitor-SD208 1 h before 2 Gy or 0 Gy of proton radiation. E. The inhibition effect of SD208 on protons treatment alone.

The DNA damage response was studied in EPC cells following 2 Gy of protons. Radiation slightly induced Smad2 phosphorylation and the expression of pSmad2 was marginally increased when compared to cells pretreated with TGFβ1 alone at early time point. An essential DNA damage response protein is p53, thus expression of phospho-p53 (Ser15) was assayed in the nuclear extract of esophageal epithelial cells at various time points. In non-irradiated cells a basal level of phospho-p53 was detected in EPC cells (Fig. 1B), while 1 hour post radiation the cells produced more phospho-p53 with a clear degradation after 8 hours. The rapid activation of p53 was ATM kinase dependent, as treatment with an ATM inhibitor led to abrogation of p53 phosphorylation (data not shown). To determine the radiation sensitivity to protons, clonogenic survival assay was performed in EPC cells (Fig. 1C). The cell survival curve obtained from protons was similar as found for gamma rays.

**Figure 4:**
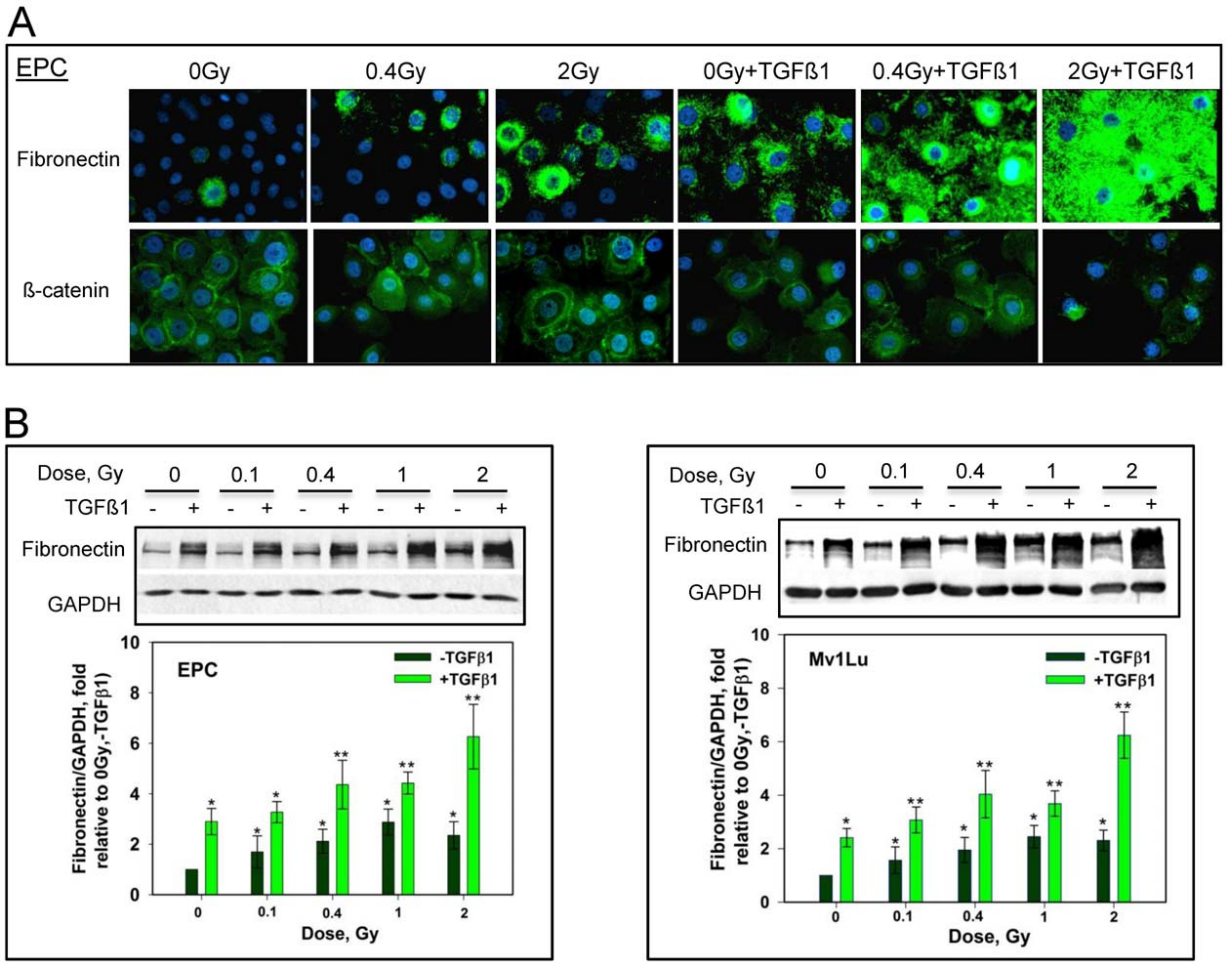
Protons can enhance TGFβ1 induced EMT-like changes in both EPC and Mv1Lu cells in a dose-dependent manner. A. Immunostaining with fibronectin and β-catenin in EPC cells upon TGFβ1 stimulation post exposures to different doses of protons. B. Effect of radiation doses on the expression of fibronectin in the presence of TGFβ1 in EPC cells (left panel) and Mv1Lu cells (right panel). Whole cell extracts prepared by M-PER were used for western blotting. Other details were shown in Figure 1. Plotted are the expression levels of fibronectin vs. GAPDH and relative to controls (0 Gy, -TGFβ1). Two protons exposures with three gels each for a total of six western blots were analyzed. Error bars indicate standard deviations. Single asterisks indicate that fibronectin/GAPDH is highly significantly difference (p-value<0.01) compared to 0 Gy un-treated cells. Double asterisks indicate that there is a highly significant difference compared to 0 Gy + TGFβ1 (p-value<0.01).

### EMT phenotypes were observed both in Mv1Lu and EPC cells in the presence of TGFβ1

The role of TGFβ1 in inducing EMT was studied two days or three days following TGFβ1 exposure in Mv1Lu cells and EPC cells, respectively. EMT was determined by alteration of cell morphology, and changes in EMT markers using immunostaining and immunoblotting. Notably, upon TGFβ1 treatment, inhibition of cell proliferation was observed in both cell lines; cells lost cell-cell contacts and changed from a compact epithelial morphology to spindle-shaped cell morphology, with significant asymmetry (Fig. 2A). To confirm that these morphological changes corresponded to EMT, markers for epithelial and mesenchymal cells, such as β-catenin, vimentin and fibronectin were detected by indirect immunofluorescence. Treatment with TGFβ1 induced mesenchymal cell markers, including an increase in fibronectin, and vimentin, and a decrease in E-cadherin and β-catenin (Fig. 2A). These changes were present when assayed by western blotting using the same antibodies both in cytoplasmic and nuclear extracts (Fig. 2B). The expression of fibronectin increased 24 hours after TGFβ1 treatment, consistently presenting after 72 hours (Fig. 2C), which can be efficiently abrogated by the pretreatment of TGFβR1 inhibitor (Fig. 2D).

**Figure 5:**
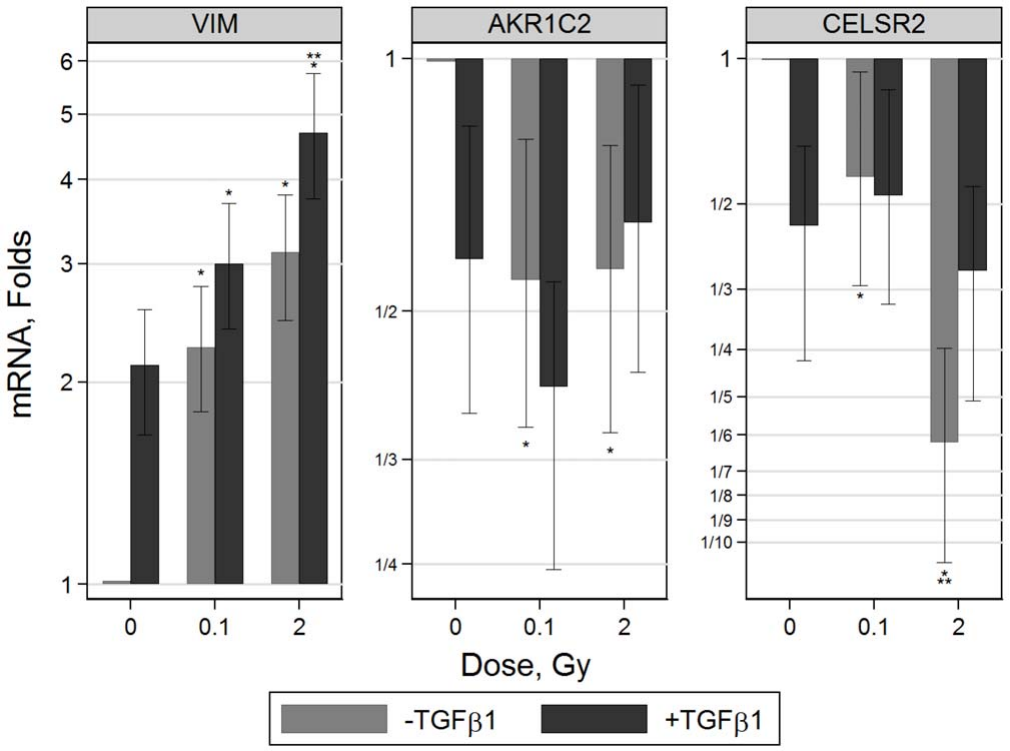
Alterations of gene expression related to EMT in proton-irradiated EPC cells, with or without TGFβ1 treatment. mRNA level of *VIM* was significantly up-regulated 3 days after treatment with TGFβ1, and a further increase was achieved when cells were irradiated with protons in the presence of TGFβ1, in a dose-dependent manner. *AKR1C2* and *CELSR2* were down regulated in single or double treated cells. Shown are results from two independent proton experiments with 6 real-time PCR measurements in total. Single asterisks indicates that there is significant difference (p-value<0.05) compared to 0 Gy untreated cells. Double asterisks indicate that the mRNA level following 2 Gy of protons is significantly different compared to 0.1 Gy. Error bars represent 95% CI intervals.

### Proton radiation can enhance TGFβ1 induced EMT in epithelial cells

To study how exposure to protons may affect EMT induction, Mv1Lu and EPC cells were exposed to protons in the presence or absence of TGFβ1. Upon 2 Gy of protons radiation, typical EMT-like morphology changes were observed in EPC cells, and more cells showed characteristic fibroblast-like morphology when pretreated with TGFβ1 (Fig. 3A). In addition, the cells lost their junctions and exhibited an even distribution when seeded at lower densities. As expected, TGFβ1 treatment resulted in alterations of EMT markers, including fibronectin, E-cadherin and β-catenin (Fig. 3B) revealed by immunofluorescence. Protons alone also induced changes similar to those observed in TGFβ1 treated cells, with an additive effect when exposed to both agents noted by the tracks of fibronectin throughout the culture flasks (Fig. 3B, right panel). Similar to the TGFβ1 alone treatments, immunoblotting revealed a difference in the expression levels of EMT markers in cells as compared to immunofluorescence (Fig. 3C). More importantly, this enhancement of EMT phenotype by radiation was abolished expeditiously via pretreatment of TGFβR1 inhibitor-SD208 (Fig. 3D and 3E).

**Figure 6:**
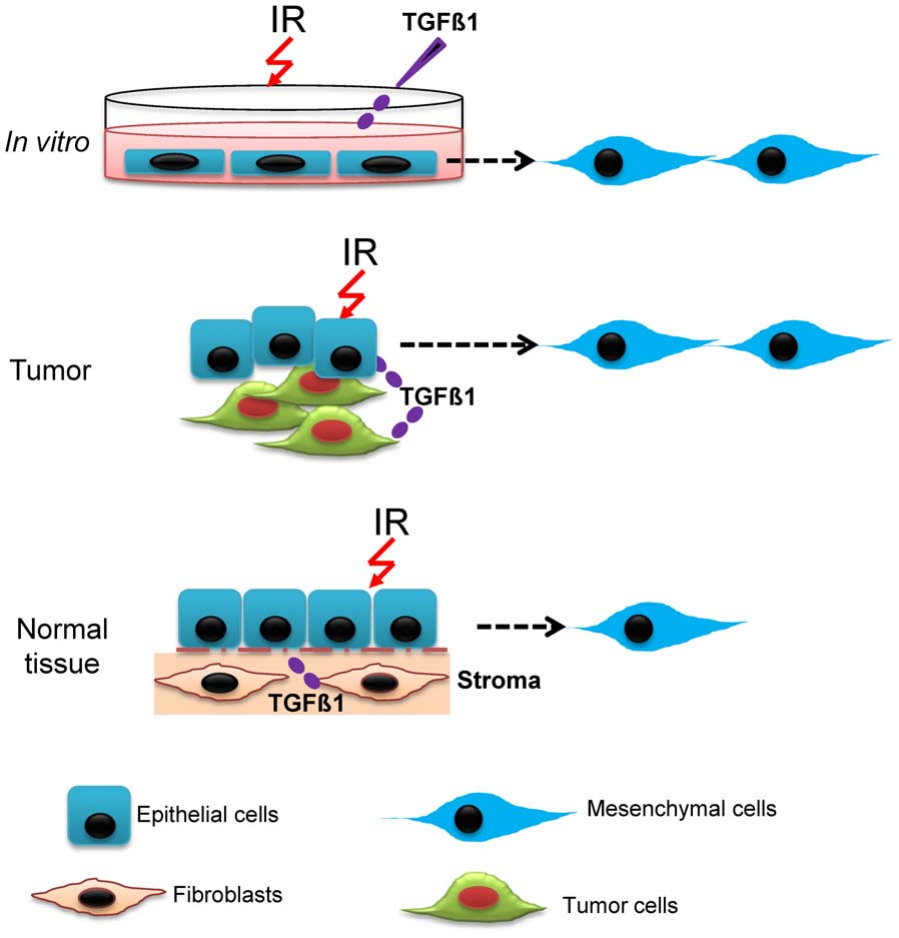
Schematic illustration of EMT induction in monolayer cell culture, cancerous tissue and normal tissue following proton irradiation. EMT occurs with different frequency and depending on the context of system studied. High levels of activated TGFβ1 *in vitro* or in cancerous tissue, enhance EMT after proton irradiation. Also the presence of other mutations such as p53, EGRF, Ras, *etc*. likely presents different mechanisms of EMT. In contrast for normal tissues, TGFβ1 is activated only at physiological levels at varying times, leading to a stochastic induction of EMT following proton irradiation.

### Elicitation of EMT enhanced by proton radiation is dose-dependent

To determine the dose-dependence of the ability of protons to induce EMT, cells were exposed to doses ranging from 0.1 Gy to 2 Gy. Immunostaining reveals a significant increasing of fibronectin signals as compared to TGFβ1 treatment alone, even at a relatively low dose of 0.4 Gy, when EPC cells are dually treated with TGFβ1 and proton radiation (Fig. 4A). Further induction is noted by following 2 Gy exposure. A slight dose dependence of fibronectin induction is observed in immunoblotting following different doses of protons. Figure 4B demonstrates a dose-dependent EMT induction from 0.1 Gy to 2 Gy when combined TGFβ1 and proton radiation in both EPC cells (left panel) and Mv1Lu cells (right panel).

### EMT related genes were altered in IR or IR and TGFβ1 treated esophageal epithelial cells

EMT related genes and transcription factors were assayed for in EPC cells using real-time PCR (Fig. 5). Of the genes tested only *VIM* (vimentin), *AKR1C2* (aldo-keto reductase), *CELSR2* (cadherin, EGF LAG seven-pass G-type receptor 2) were significantly changed in a consistent manner. mRNA levels of *VIM* increased two-fold after a three-day TGFβ1 exposure in non-irradiated cells. The same magnitude of effect was achieved upon 0.1 Gy of protons alone. However, when TGFβ1 and radiation treatments were combined *VIM* levels increased significantly in a dose-dependent manner. Genes known to be down-regulated during EMT such as *AKR1C2* which encodes for a 3a-hydroxysteroid dehydrogenase, and *CELSR2* were assayed for. Both exhibited reduced levels in cells treated with TGFβ1 alone, radiation alone or a combination of TGFβ1 and proton radiation.

## Discussion

These studies were initiated to investigate the potential for protons to induce epithelial to mesenchymal transition in mammalian cells. Most studies of EMT use carcinoma cell lines, while our focus was to consider possible normal tissue effects. To examine this possibility, both non-transformed Mv1Lu and hTERT immortalized human esophageal epithelial cells (EPC) were exposed to protons, both in the presence and absence of TGFβ1, and EMT induction was assayed for by immunofluorescence and western blotting. We observed that either TGFβ1 or proton treatment alone could stimulate EMT in both cell lines, but additionally that protons could also enhance TGFβ1-mediated effects. The EMT phenotype was established by standard morphological alterations including loss of cell-cell contact, formation of spindle shaped cells, and loss of epithelial markers and gain of mesenchymal cell markers. Mv1Lu cells, an established cell line for studying TGFβ1/Smad signaling [16], also exhibited EMT in the presence of TGFβ1 as well as proton irradiation. Similar to Mv1Lu cells, EMT-like changes, including the down-regulation of cadherins, translocation of β-catenin, and abundant expression on fibronectin and vimentin were observed in TGFβ1 treated esophageal epithelial cells. These TGFβ1 induced effects were enhanced by proton exposure. In addition, we observed a mild induction of EMT like features with protons alone.

Notably, our study has revealed that protons alone or in combination with TGFβ1 treatment can induce EMT associated changes in normal human epithelial cells. EMT has been linked to radiation-induced fibrosis, one of the most common late effects of radiotherapy. This finding has also been noted in human mammary epithelial cells exposed to gamma-rays and heavy ions where radiation can sensitize cells to undergo TGFβ1-mediated EMT [17], and in lung, colon, or mammary carcinoma cells exposed to high doses (2 Gy) of gamma-rays [18]. In the current study, the dose dependency of this effect was investigated, and surprisingly very low proton doses (0.1 Gy) were shown to induce an EMT effect in the presence of TGFβ1. This low dose effect is significant given: 1) during proton radiotherapy, although energy is mostly deposited in tumor tissue, cells located at the border of tumor will absorb lower doses of protons often at a lower energy; 2) protons are major components of space radiation exposures [4]. Although the total body dose received in space is moderately low compared to what is received in radiotherapy, these studies reveal that very low doses of protons may still elicit EMT and result in potentially detrimental effects.

Studies [12], [13] have revealed many solid tumors produce high levels of TGFβ1, which can be activated by environmental stimuli such as radiation. Once activated TGFβ1 may enhance the risk or accelerate EMT and based on reported observations [19]–[21]. Moreover, recent studies note that tumor cells can gain cancer stem cell properties as a result of EMT [7], [8], leading to a higher probability of metastasis and radiation/drug resistance. This finding is especially relevant given ninety percent of all malignant tumors are of epithelial origin, and many are radiation resistant. Recently, studies have pointed to the existence of a “tumor or cancer-initiating cell” providing new insights into the potential origin of cancer. In addition, recent studies suggest that cells induced to exhibit EMT are primarily multi-potent progenitor cells, with properties similar to epithelial stem cells [6].

Under experimental conditions where endogenous TGFβ1 is added in vitro to normal or carcinoma cells lines, activated TGFβ1 levels may be increased for extended periods of time. In contrast, physiological levels of activated TGFβ1 in normal tissue may vary for different times and conditions including following radiation exposure, which should modify the frequency of EMT when cells are irradiated compared to endogenous conditions (Fig. 6). The dose response we observed for proton doses from 0.1 to 2 Gy, suggest a stochastic model of EMT in normal epithelial populations, which would be dependent on the expression levels of TGFβ1 at the time of irradiation and on the radiation dose. In contrast, pre-cancerous tissues which over-express TGFβ1 would undergo a high frequency EMT following proton irradiation possibly leading to a greater propensity for metastasis.

The mechanism underlying IR-induced EMT may involve Smad signaling, Ras activation, or the Erk/MAPK signaling cascade activated by elevated TGFβ1 levels [5]–[8]. Several other oncogenic pathways, such as peptide growth factors, Src, integrin, Wnt/β-catenin and Notch may also induce EMT in different cellular contexts [22], [23]. The increased gene expression found in our experiments with protons was significant only for *VIM*, *AKR1C2* and *CELSR2*. In contrast previous studies by others with esophageal epithelial cells over-expressing EGFR or mutant for p53 without irradiation led to distinct gene expression changes [29]. Alternatively, the mechanism may involve exosome-mediated cell-cell communication, resulting in increased anti-apoptotic signals post proton radiation [24], and protection of tumor cells from programmed cell death, tumor radioresistance and invasion. However, apoptotic tumor cells were found to release membrane-bound TGFβ1 [25]; and others [26] have noted that the surface TGFβ1 from cancer-derived exosomes can initiate Smad signaling. Although these studies are suggestive, the molecular basis of radiation-enhanced EMT and possible differences between cell culture and tissue results need to be further investigated.

Taking together, our studies suggest that proton radiation, an advanced choice for deep-seated tumor treatment, may trigger EMT, and enhance TGFβ1–mediated EMT in normal tissues, even following a single acute low dose. Similar effects with low doses of x-rays have been observed using mammary epithelial cells [17]. The many benefits in the use of proton treatment need to be weighed against the potential risks, including EMT and other potentially detrimental effects in normal tissue exposed to low doses of protons. TGFβRI inhibition can efficiently block this process, perhaps providing a guide for future therapeutic strategies in the treatment of tumors and metastasis.

## Materials and Methods

### Cell culture and chemicals

Non-transformed Mv1Lu cells were purchased from ATCC, and maintained in MEM medium supplemented with 10% FBS. hTERT-immortalized human esophageal epithelial cells (EPC, a kind gift from A.K. Rustgi) [27], [28] were cultured in keratinocyte-SFM medium supplemented with 50 µg/mL bovine pituitary extract, 1.0 ng/mL EGF, 100 U/mL penicillin, and 100 µg/mL streptomycin. Recombinant human TGFβ1 (PeproTech, Rocky Hill, NJ) was added at a concentration of 2 ng/ml for the indicated period of time. A potent TGFβRI inhibitor-SD208 [29] was purchased from Tocris Bioscience (Ellisville, Missouri). 1 µM SD208 in DMSO (0.1% v/v) or DMSO (0.1% v/v) alone were added to medium 1 hour prior to the addition of TGFβ1 as described previously [30].

### Irradiation

Experiments using 100 MeV protons slowed down using polyethylene shielding to a distribution of proton energies with a mean energy of 5 MeV and mean LET of 10 keV/µm were performed at the NASA Space Radiation Laboratory (NSRL) in Brookhaven National Laboratory (BNL, NY). A 20×20 cm beam ensured complete and equal exposure to all samples. The dose rate was between 0.25 to 1 Gy/min dependent upon dose. T25 flasks containing exponentially growing cells were exposed vertically with the cell surface facing the beams. Additional experiments with protons were performed at the MD Anderson Proton Cancer Treatment Center (Houston, TX). For these experiments, 150 MeV protons were slowed to a mean energy of 5 MeV using brass collimators with a dose-rate of 0.5 Gy/min. For both the 100 and 150 MeV beams a small percentage of the dose will be from secondary protons and other recoil nuclei [31]. Dose uniformity at BNL and MD Anderson facilities was ±5%. ^137^Cs gamma radiation experiments were completed at NASA Lyndon B. Johnson Space Center (Houston, TX). Different radiation doses as indicated were delivered 1 h after TGFβ1 treatment. Cells at subconfluence (below 50%) were used before radiation and/or TGFβ1 treatment. Cells were then harvested 2 days (Mv1Lu) or 3 days (EPC) after TGFβ1 and/or proton irradiation.

### Clonogenic cell survival assay

To evaluate radiosensitivity to protons 2×10^5^ esophageal epithelial cells were plated per T25 flask with 5 ml of medium. Cells were then irradiated 48 hours later and were collected and plated in dilutions aiming at 50–100 colonies per 60 mm dish. Triplicates were prepared for each datum point and incubated for 10 days to allow colonies to develop. Colonies were stained with crystal violet (100% methanol solution) before counting.

### Western blotting and immunofluorescence

Whole cell extracts were used to detect the expression of EMT markers by western blotting. Cells were lysed either in M-PER Mammalian Protein Extraction Reagent (Thermo Scientific, Rockford, IL), supplemented with Halt protease inhibitor cocktail (Thermo Scientific), or in RIPA buffer (50 mM Tris, 150 mM NaCl, 0.1% SDS, 0.5% NaDeoxycholate, 1%NP40, 1 mM PMSF and Halt inhibitors added freshly) as indicated. M-PER lysis buffer extracts only nuclear and cytoplasmic proteins. Nuclear extracts were prepared using NE-PER Nuclear Protein Extraction Kit from Thermo Scientific, to detect proteins presenting in the nuclei. The optical density of each band was quantified using ImageQuant software. Mouse anti-vimentin and rabbit anti-fibronectin were purchased from Sigma-Aldrich; rabbit anti-phospho-Smad2 (S465/S467), rabbit anti-phospho-p53 and mouse anti-β-catenin were from Cell Signaling Technology Inc.; mouse anti-Smad7 from R&D mouse anti-TATA Box Binding Protein 1 (TBP1) and mouse anti-Glyceraldehyde 3-phosphate dehydrogenase (GAPDH) from Millipore; and mouse anti-E-cadherin from BD Biosciences.

For immunofluorescence, cells were grown on LabTek 8-well chamber slides, and fixed with 4% paraformaldehyde, followed by treatment with ice-cold methanol for 10 min. After permeabilization with 0.5% Triton X-100 in PBS for 3 min, cells were blocked with 10% normal goat serum and incubated with indicated primary antibodies. Detection was accomplished using Alexa Fluor 488 or Fluor 594 conjugated secondary antibodies and nuclei were counterstained with 4, 6-diamidino-2-phenylindole (DAPI). Immunofluorescence was evaluated with a fluorescence microscope Axioplan2 (Zeiss, Sweden).

### RNA isolation, reverse transcription and quantitative real-time PCR

Total RNA was extracted using RNeasy Mini kits (Qiagen Inc., Valencia, CA) and its concentration determined using Shimadzu Spectrometer. cDNA was synthesized using 500 ng of total RNA in a 20 µl reverse transcriptase reaction mixture from QuantiTect, and a CRF96 real-time PCR machine (Bio-Rad, Hercules, CA). Custom- designed microarrays with incorporated primers were used for real-time quantitative PCR (SABioscience, a Qiagen Company).

### Statistical analysis

Quantitative real-time PCR data was analyzed using t-test with bonferroni correction. Differences with *p*<0.05 are considered significant.
